# The Impact of COVID-19 Lockdown on People With Epilepsy and Vagal Nerve Stimulation

**DOI:** 10.3389/fneur.2021.640581

**Published:** 2021-02-26

**Authors:** Eleonora Grande, Tommaso Tufo, Marco Ciavarro, Ines Di Muccio, Filomena Fuggetta, Martina Silvestri, Giuseppina Bevacqua, Jacopo Lanzone, Giovanni Assenza

**Affiliations:** ^1^Department of Neuroscience, Imaging and Clinical Sciences, University “Gabriele d'Annunzio” of Chieti-Pescara, Chieti, Italy; ^2^Neurosurgery, Policlinico A. Gemelli Foundation Istituto di Ricovero e Cura a Carattere Scientifico, Catholic University, Rome, Italy; ^3^Neuromed for Istituto di Ricovero e Cura a Carattere Scientifico, Pozzilli, Italy; ^4^Neurosurgery, A.O.S.G Moscati, Avellino, Italy; ^5^Department of Human Neurosciences, “Sapienza” University of Rome, Rome, Italy; ^6^Neurology, Neurophysiology and Neurobiology Unit, Department of Medicine, Università Campus Bio-Medico di Roma, Rome, Italy

**Keywords:** epilepsy, vagal nerve stimulator, COVID-19, mental health, telemedicine

## Abstract

**Objectives:** Restrictive measures adopted during the COVID-19 pandemic, in order to limit contagion, have had a severe impact on mental health. The burden of lockdown has been particularly heavy on patients with chronic neurologic diseases such as People with Epilepsy (PwE). Our survey aims to describe the struggles and needs of Drug-Resistant (DR) PwE with implanted Vagal Nerve Stimulator (VNS) during the first wave of the COVID-19 lockdown in order to find strategies that help patients cope with present or future periods of restriction.

**Methods:** We collected answers from 30 respondents who underwent an online survey including socio-demographic and clinical information and COVID-19-related information. Depression, anxiety symptoms, and sleep quality were investigated in patients through BDI II, GAD-7, and the PSQI scale.

**Results:** In all, 46% of our sample reported an increase in the number of seizures; the entire sample complained of epilepsy-related issues (medication availability, VSN adjustments, anxiety, sleep disturbance); one out of three participants reported major epilepsy issues felt urgent; 30% had to postpone scheduled examination. Significantly higher scores for depression and anxiety scales were found in patients who perceived seizure frequency worsening and reported major epilepsy-related issues.

**Conclusion:** Preliminary findings showed that the first lockdown influenced the clinical and psychological status of PwE and was related to seizures worsening. The lack of medical assistance and control on VNS therapy left patients to cope with the situation without a chance to contact a specialist. We discuss how a wider implementation of telemedicine programs could facilitate remote assistance of PwE with a VNS implant.

## Introduction

Coronavirus infection (COVID-19) rapidly spread worldwide during the early months of 2020 (1) to the point of being declared a pandemic by the World Health Organization (WHO) in March. Early on, imposing lockdown measures was the most widespread strategy to limit the diffusion of the disease, alleviating the burden on healthcare systems. Restrictive measures severely affected the social and mental health of individuals, and thus, lockdown was associated with an increase in mental health-related issues, such as anxiety, depression, and sleep disorders, as was shown by a national cross-sectional study on the general Italian population (2). Patients with chronic neurologic diseases suffered even more from the hardships of lockdown (3–6). Restrictive measures imposed during the first wave of COVID-19 caused a decrease in availability of neurological assistance in Italy (5). This affected, among others, PwE, given that most healthcare services or hospitals were not ready to implement telemedicine, which had been proposed as a potential solution during the time of restrictions (7). In particular, more frail patients, such as DR-PwE with implanted VNS, were not able to attend scheduled visits fundamental to titrate stimulation's parameters to optimize efficacy and tolerability. VNS is implanted in DR-PwE as palliative therapy to reduce seizure frequency, when pharmacological and surgical approaches fail (8, 9), and once implanted, it repeatedly needs to be regulated, to slowly reach the target stimulus intensity, possibly with minimal adverse effects (10). Adverse events (mainly hoarseness, cough, paresthesia during the ON phase of VNS) can be easily overcome by frequency, duration, and intensity adjustments. Unfortunately, VNS devices cannot be remotely controlled but require a physical intervention of the clinicians. For these reasons, VNS patients need continuous and cadenced follow-up visits, which are difficult to guarantee during the COVID-19 lockdown. Since VNS patients carry an implanted device upon which they do not have direct control, they are vulnerable to the reduced availability of neurological assistance during lockdown. Thus, we designed a survey targeting PwE patients with VNS. With this study, we aimed at identifying and discussing the special needs of PwE with VNS implant during periods of reduced availability of follow-up and propose helpful strategies to implement during this new wave of contagion.

## Materials and Methods

Data were collected from PwE with implanted VNS attending the outpatient epilepsy clinics in two major hospitals in Rome (Italy), the “Policlinico Universitario Fondazione Agostino Gemelli—Roma” and “Policlinico Universitario Campus Bio-Medico di Roma.” All patients gave their consent to be contacted for research purposes at the moment of their hospitalization for VNS surgery.

**Figure 1:**
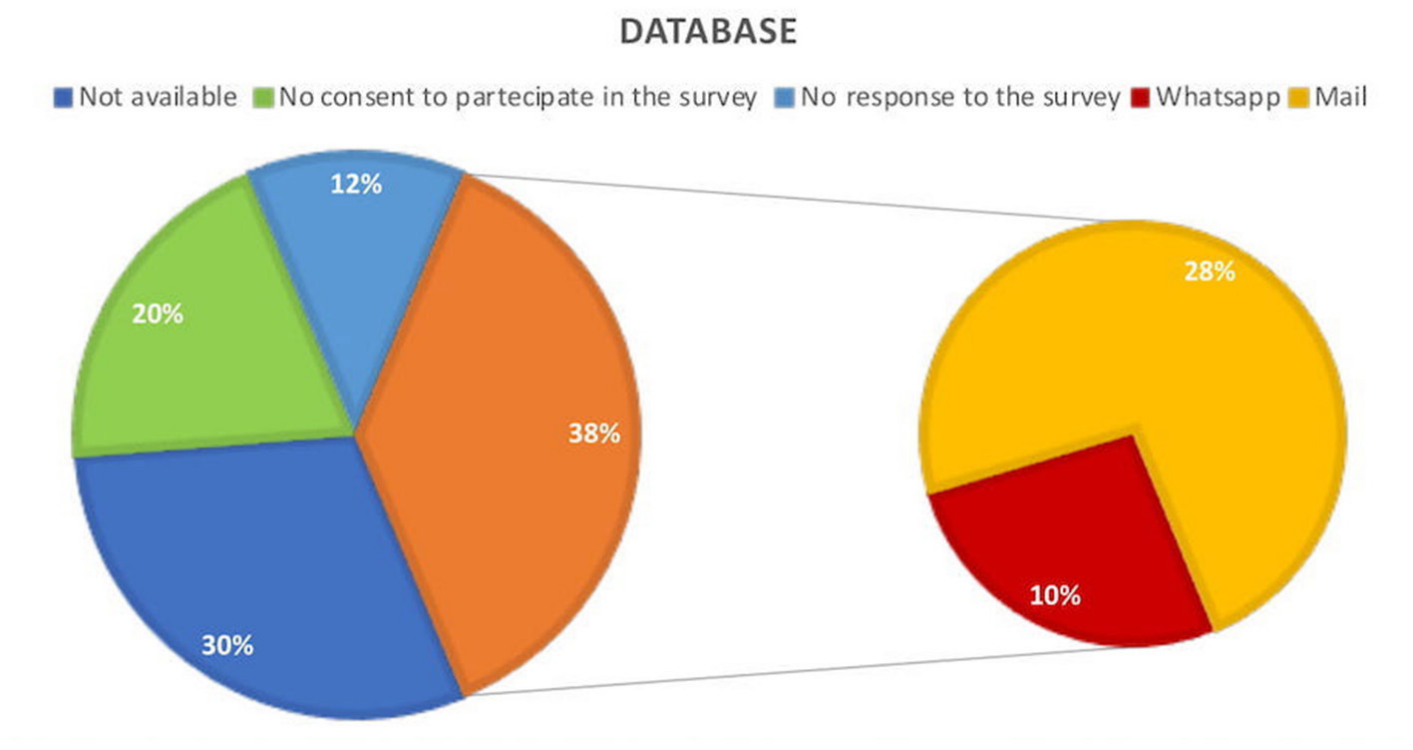
Participants in the joint database who consent to take part in the survey and those who did not; reception of the online questionnaire.

Experimenters contacted all PwE with implanted VNS (by phone) from the joint database to assess their consent to receive an online questionnaire before it was sent to patients and their caregivers through mail or WhatsApp® contact. Figure 1 shows the database features. The inclusion criteria involve an implanted VNS device regardless of the etiology and consent to take part in the study; the exclusion criteria involve duplicated answers (i.e., participants with the same birth date answer twice) and unreliable answers.

The survey was performed during the months of April and May 2020, which was at the maximum of the Italian contagion curve. Given the neurocognitive impairment experienced by many PwE with VNS implant, if the patient was not able to independently answer our survey, the caregiver provided the response with clinical information, referring to the patient. The questionnaire contains a brief description of the study. They electronically consented to complete the questionnaire after reading an informed consent on their devices, and data were stored anonymously. Clinical information on epilepsy, such as seizure frequency and type, anti-seizure medications (ASMs), and duration of the disease, was collected. In addition, participants were asked COVID-19-related information (symptoms, fever, hospitalization) and VNS-related questions (the seizure frequency reduction and seizure intensity reduction induced by VNS). Participants had to accurately report the number of monthly seizures occurring in the 50 days during the first lockdown (referred to as the “COVID-19 period”) and in the 50 days before March 11 (start of the first lockdown in Italy; referred to as the “pre-COVID-19 period”). Most patients and their caregivers have a long-standing story of epilepsy, are accustomed to their subjective semiology, and are instructed in identifying symptoms. They keep a daily seizures diary; thus, their report is considered to be reliable. Finally, participants were asked information on major (i.e., clinical issues were felt to be urgent with the inability to contact a specialist; clinical problems that in normal situations would make the patient look for a specialist's opinion) and minor complications (i.e., ASM availability, VNS adjustment, anxiety, and sleep disturbance) due to COVID-19 restrictions.

Mental health status was investigated only when the respondent was the PwE in person. We used the Beck Depression Inventory scale II (BDI-II) (11), a 21-item scale (scored 0–3), as a self-report measure of common depressive symptoms. Anxiety symptoms were assessed through use of the General Anxiety Disorder-7 (GAD-7) (12). Pittsburg Sleep Quality Index (PSQI) (13) was administered to evaluate sleep quality. Previous items (clearly showed in Supplementary Table 2) were included in an online survey questionnaire created using the free open-access Google™ Forms (https://www.google.com/forms/about/) application, as in a prior study on COVID-19 consequences (6). Data were treated according to the European regulation GDPR n. 2016/679. Our ethics committee was involved in the development of the study, and the local Ethics committee was officially notified of the study as a prospective observational study with anonymous data sampling. Proper ethical committee approval was not necessary for this type of study.

We applied this protocol in an emergency phase of the COVID-19 pandemic with the hope that the preliminary data received, in the future, could be expanded through national and international collaboration to better depict the needs of the special population of VNS PwE.

### Statistical Analysis

We performed statistical analysis using R studio software 1.3.1. The Kolmogorov-Smirnov test was used to test the normal distribution of continuous variables. Normally distributed data are reported as Mean ± SD, and their differences are described with a Student *t*-test; nonnormally distributed data are reported as Median and Interquartile Range (IQR), with their differences analyzed by Wilcoxon's test. The alpha level was set at 0.05 for statistical significance.

## Results

### Demographic and Clinical Data

We collected responses to the questionnaire from 30 participants. The whole sample came from central/southern Italy. The sample comprised 30 PwE (13 patients and 17 caregivers), with 11 females of a mean age of 45.6 ± 13.8. The whole sample has an implanted VNS in the active phase. Demographic information was displayed in Table 1 while education, marital, and working status are reported in Supplementary Table 1. Table 1 also includes epilepsy clinical data, such as etiology, ASM, types of seizures, and non-pharmacological therapies. All the patients were under ASMs, and only 3 (10%) of these did not receive a neurological examination during the last year. Furthermore, Table 1 reports data concerning the number of seizures that occurred during the 3 months prior to the interview, specifically, the number of all seizures (focal and tonic-clonic generalized seizure, median number 38.5, IQR 110.7) and the number of generalized tonic-clonic seizures exclusively (median number: 1.5, IQR: 20). Moreover, the number of seizures before and during the COVID-19 period were registered. Finally, Table 1 includes information about the benefit of VNS and outcomes of VNS with respect to seizure frequency and intensity.

**Table 1:** Demographic and clinical data of the whole sample.

**Patients**	**Epilepsy etiology**	**Surgery**	**Year**	**Sex**	**Years of epilepsy**	**ASM number**	**Specific ASM**	**Years VNS**	**Type of seizures**	**Non Pharmacological therapies**	**N of seizures (3 months)**	**N of tonico-clonic seizures (3 months)**	**N of seizures pre Covid-19**	**N of seizures during Covid-19**	**VNS benefit**	**VNS N of seizures reduction**	**VNS % frequency reduction**	**VNS intesity reduction**
1	Unknown		58	M	11	3	CBZ TPM PER	12	FS FBTC		12	0	8	4	Yes	Yes	>50%	Yes
2	Unknown		46	F	12	3	LEV CBZ PB	8	FS FBTC		178	5	91	133	Yes	Yes	>25%	Yes
3	Structural		45	F	10	3	ZNS CBZ PB	7	GTC		87	0	34	44	N/A	N/A	N/A	N/A
4	Structural		52	M	20	4	CBZ VPA LEV PER	22	FS FBTC		15	0	8	9	Yes	Yes	>75%	Yes
5	Genetic		25	F	24	2	VPA CBZ	15	FS FBTC	Ketogenic diet	45	6	28	28	Yes	Yes	>75%	Yes
6	Unknown		71	M	36	2	LEV LTG	10	AA GTC		16	0	12	23	No	Yes	<25%	Yes
7	Unknown		44	M	44	3	OXC ZNS PB	8	GTC T		32	20	34	26	Yes	Yes	>50%	No
8	Unknown		51	M	21	2	VPA BRV	12	GTC		6	0	2	1	Yes	Yes	<25%	Yes
9	Unknown		56	F	12	3	ZNS LAC BRV	11	GTC		60	0	50	30	Yes	Yes	>50%	No
10	Unknown		46	M	18	4	TPM PER CBZ LTG	9	FS FBTC		181	0	50	70	N/A	N/A	N/A	N/A
11	Structural		34	M	3	3	PB RAC BRV	11	FS FBTC		30	0	14	16	Yes	Yes	>50%	Yes
12	Unknown		51	M	1	2	CBZ GBP	12	GTC		7	3	2	5	Yes	Yes	<25%	Yes
13	Unknown		56	M	25	3	OXC PER LAC	14	FS FBTC		13	13	7	7	N/A	N/A	N/A	N/A
14	Unknown		38	M	1	2	CBZ PB	15	GTC		275	90	155	147	Yes	Yes	>50%	Yes
15	Anterior Temporal Lobectomy	Yes	29	M	1	2	OXC FBN	15	FS FBTC		90	30	30	30	Yes	Yes	>25%	No
16	Unknown		38	M	5	4	CBZ ZNS TPM CLN	13	FS FBTC		N/A	N/A	N/A	N/A	No	No	<25%	No
17	Unknown		76	F	24	1	VPA	14	FS FBTC		3	0	2	3	Yes	Yes	>75%	Yes
18	Unknown		41	F	6	3	VPA FBN CBZ	16	GTC		100	20	60	45	Yes	Yes	<25%	Yes
19	Unknown		34	M	8	3	OXC CBZ PER	10	FS FBTC		4	12	8	6	Yes	Yes	>25%	Yes
20	Anterior Temporal Lobectomy	Yes	27	F	0	3	VPA CBZ PER	4	GTC		120	0	40	60	No	No	<25%	No
21	Unknown		44	M	44	3	OXC PB ZNS	13	FS FBTC		32	27	24	26	No	Yes	<25%	Yes
22	Unknown		23	M	7	3	VPA CBZ PB	3	GTC AA	Ketogenic diet	500	150	45	50	No	No	N/A	No
23	Unknown		30	M	1	4	FBN OXC TPM CLN	5	FS FBTC		400	12	60	135	No	No	<25%	No
24	Genetic		69	F	29	3	TPM PB SL	29	FS FBTC		3	3	4	5	Yes	Yes	>90%	Yes
25	Unknown		55	F	13	3	ZNS LAC BRV	12	GTC		60	0	33	33	Yes	Yes	>90%	Yes
26	Unknown		50	M	21	2	VPA BRV	11	GTC		10	23	1	1	Yes	Yes	>25%	No
27	Structural		44	M	5	4	CBZ LTG ZNS CBZ	22	FS FBTC		4	0	2	2	N/A	N/A	N/A	N/A
28	Unknown		55	F	4	3	CLN CBZ PB	15	FS FBTC		N/A	N/A	N/A	N/A	Yes	Yes	>50%	Yes
29	Unknown		56	F	1	5	TPM BRV LAC PER CBZ	12	FS FBTC		48	0	20	7	Yes	Yes	>25%	No
30	Structural		25	M	6	3	LEV PER CLN	16	GTC	Ketogenic diet	120	20	80	45	Yes	Yes	<25%	No

*GTC, Generalized tonic-clonic seizures; FS, focal seizures; FBTC, focal to bilateral tonic-clonic; AA, atypical absence; T, tonic seizures; N/A, no answer; CBZ, Carbamazepine; ESL, Eslicarbamazepine; OXC, Oxcarbamazepine; VPA, Valproic Acid; ZNS, Zonisamide; TPM, Topiramate; LEV, Levetiracetam; BRV, Brivaracetam; CLN, Clonazepam; CBZ, Clobazam; PB, Phenobarbital; LTG, Lamotrigine; LAC, Lacosamide; PER, Perampanel; FBM, Felbamate*.

### COVID-19 Data

Symptoms of COVID-19 infection were specifically investigated. Two participants reported fever, and two underwent a nasopharyngeal swab test for SARS-CoV-2 (no positive and no hospitalization).

### Epilepsy During the COVID-19 Lockdown

#### Seizure Number Report

For the whole sample preliminary findings showed no significant difference in the number of seizures during the pre-COVID-19 period and COVID-19 pandemic: the median number of seizures during the COVID-19 period was 26 (IQR 39.75) and 26 (IQR 41.5) during the pre-COVID-19 pandemic period (*p* > 0.05).

#### Epilepsy-Related Issues

**Figure 2:**
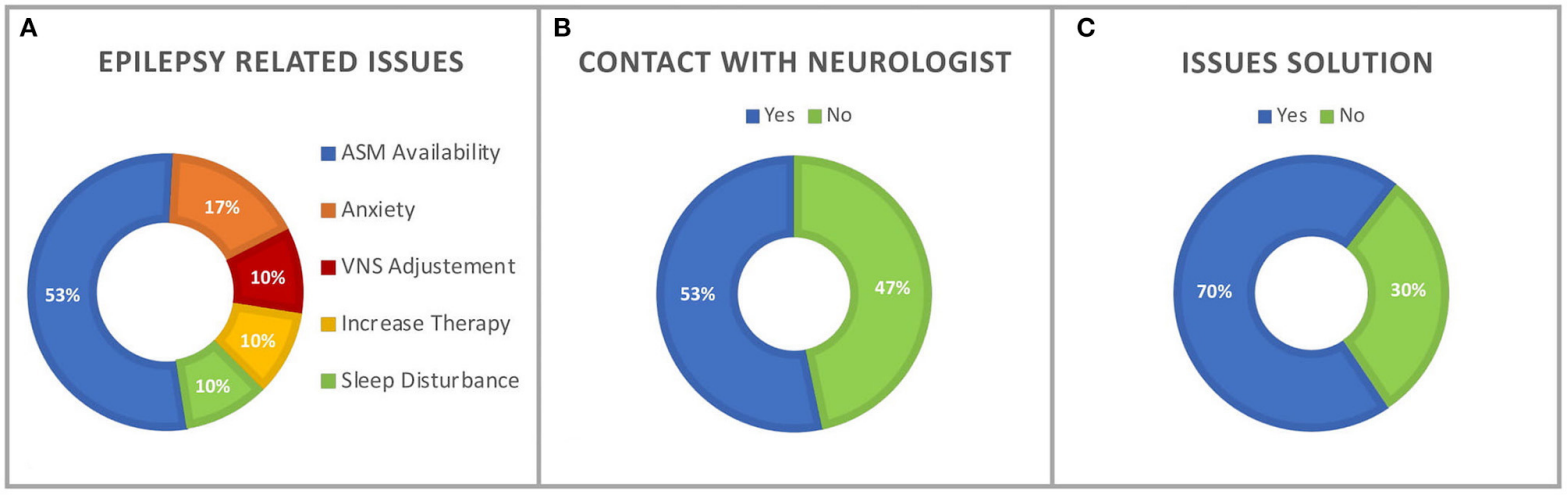
**(A)** Percentage of epilepsy-related issues experienced during the COVID-19 period. **(B)** Percentage of participants who reached a neurologist during the COVID-19 period. **(C)** Percentage of participants who managed to solve epilepsy-related issues.

Due to COVID-19 restrictions, eight participants of the whole sample (27%) reported major epilepsy-related issues, while all participants reported minor problems as displayed in Figure 2A. Figure 2B highlights that 16 participants (53%) achieved to get in touch with their neurologist during the COVID-19 period (9, 56% personal mobile calls; 4, 25% short text messages/WhatsApp messages; 2, 16% mail; 1, 6% doctor's office calls). Ten patients (33%) had to postpone a scheduled medical visit, and nine participants (30%) did not manage to solve their problems (Figure 2C). No patient was hospitalized for epilepsy-related problems.

### Psychometric Assessment

In the last section of the questionnaire, anxiety, depressive symptoms, and sleep quality were investigated in the patients' group (*n* = 13). The BDI II median score was 6 (IQR 13.5), with three patients (23%) reporting out of normal range values. The GAD-7 median score was 5 (IQR 4.5), and seven patients (54%) reported out of normal range value. The PSQI index median score was 4 (IQR 5), with five patients (38%) showing out of normal range scores.

**Figure 3:**
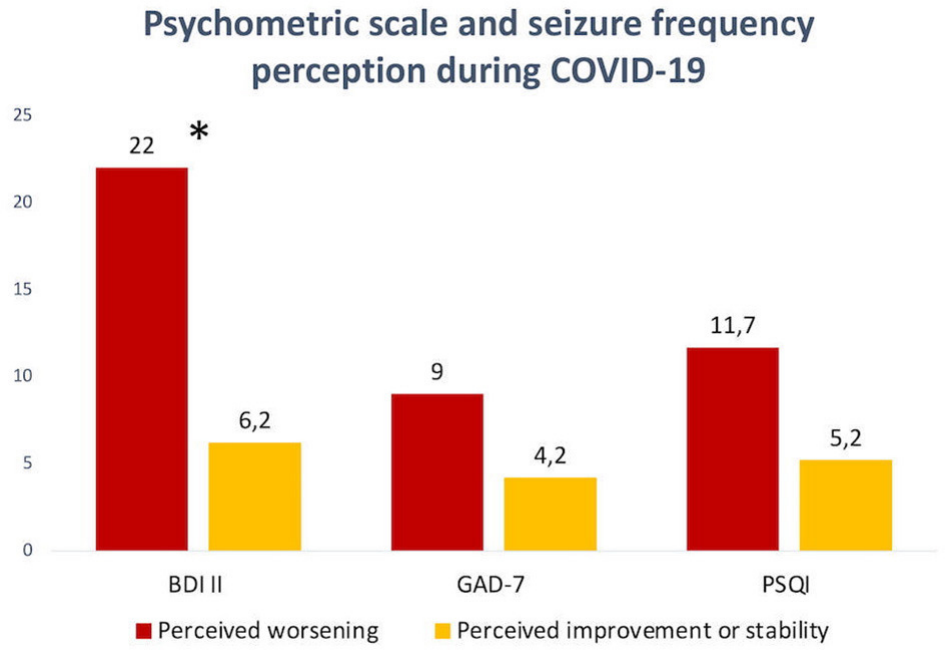
Differences in psychometric scales scores between patients who perceived seizure frequency worsening during the COVID-19 period and patients who perceived seizure frequency stability or improvement. Significant difference in the BDI II scale value (*p < 0.05).

Our preliminary results exhibited a significant difference (*p* < 0.05) on the depression scale between patients who perceived an increase in seizure frequency (3, 23%; BDI II: 22 ± 26) and patients who reported reduction or stability (10, 76%; BDI II: 6.2 ± 7.49) (Figure 3). These exploratory results suggest higher depressive symptoms in patients who perceived seizure frequency worsening during the COVID-19 period.

**Figure 4:**
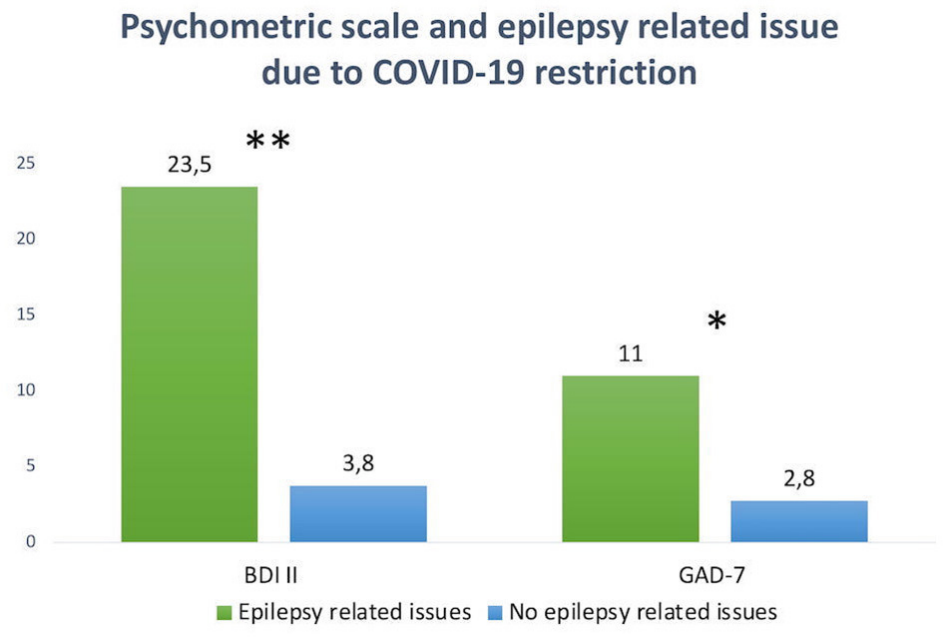
Significant differences in BDI II scale value (**p < 0.01) and GAD-7 value (*p < 0.05) between the group who experienced major epilepsy-related issues during the COVID-19 pandemic and the group who did not.

To address major epilepsy-related issues during the COVID-19 period, a prior comparison between the group who experienced major epilepsy-related issues (4, 31%) and those who did not (9, 69%) was performed. In the group with epilepsy-related issues during the COVID-19 period, the BDI II score (23.5 ± 19.89) and GAD-7 score (11 ± 7.11) were significantly greater (*p* < 0.01; *p* < 0.05, respectively) (Figure 4) than the group who did not report major epilepsy-related issues (BDI II: 3.8 ± 4.6; GAD-7: 2.8 ± 1.78), indicating increasing symptoms of anxiety and depression in the group who experienced global epilepsy-related adverse event due to COVID-19 restrictions.

## Discussion

We designed the present survey to explore the impact that the COVID-19 lockdown had on the PwE with VNS implant, one of the frailest group of patients with epilepsy. During COVID-19, a recent wider survey on PwE evidenced that they face many difficulties. Thus, we expected that PwE with VNS, that need more frequent medical examinations, might also face major clinical issues.

### COVID-19 Lockdown Impact on Epilepsy

The whole sample has an implanted VNS in the active phase, and about half of the participants report a benefit in seizure frequency > 50%, as commonly presented in the literature (14). According to our preliminary findings, we did not find significant changes in seizures during the COVID-19 lockdown. On the other hand, up to a third of respondents encountered major disease-related issues during the lockdown period. The entire sample complained of minor issues, such as postponed scheduled medical visits, reduced ASM availability, need of VNS adjustments, anxiety, and sleep disturbance. Nearly half of the respondents could not contact a specialist, leading to a third of them not being able to find a solution to major or minor complications. These data closely replicate previously reported findings from a larger Italian survey (6), confirming that restrictive measures of the first wave of COVID-19 resulted in a medical care reduction for PwE and also insufficient service in the antiseizure medications supply chain. Furthermore, it is worth mentioning that our population often reported specific problems with VNS devices that are not solvable without the assistance of specialists, thus exposing PwE with VNS to a significant discomfort and clinical risk during lockdown. These data underscore the urgency of the implementation of remote assistance with VNS devices, in order to reduce malfunctions or to provide specialistic assistance and to help patients to alleviate adverse events in case of isolation.

### Epilepsy and Psychometric Reports

Early results of psychometric scales showed that half of the patients reported abnormal values on the anxiety symptoms scales, and one out of four showed abnormal values in the assessment of depressive symptoms. Poor sleep quality was observed in 4 out of 10 PwE. Prior depressive and anxiety symptoms emerged among our PwE with VNS sample confirmed the previous results in a larger PwE sample and seems strictly related to both a reported increase in seizure frequency and the presence of epilepsy-related issues. Our preliminary findings showed that PwE reporting worsening of seizures and epilepsy-related issues had worse depressive and anxiety symptoms than PwE who did not.

The format of our questionnaire cannot proove the directionality of the relationship among seizure worsening, depressive/anxiety symptoms, and clinical issues complaints; we nonetheless want to underscore the need for assistance of these frail patients. One interpretation of our preliminary results is the hypothesis that the combination of epilepsy-related issues and seizure worsening might have negatively influenced the psychological status of PwE. However, we cannot rule out the possibility that the reported worsening could be the result of poorer scores in depression and anxiety scales affecting self-perception and the perception of each respondent's own disease. In fact, PwE often demostrate incongruence confronting to their own disease consistently with the fact that patients with long-standing epilepsy are often found to be alexithymic, meaning they have scarce insight into their condition both physical and psychological (15).

It is not surprising that more severe patients, such as those PwE with VNS, exposed to factors directly influencing quality of life, such as the perception of stigma and the number of medications (15, 16), report more anxiety and depression. Thus, our purpose is to highlight the need for assistance in PwE with VNS, in whom depression is very frequent comorbidity and who constantly have to cope with the anxiety derived from having an implanted device that they cannot directly control, along with the fear of unfathomed adverse effects or of the abrupt stop of the titration plan during the lockdown period. For many of them, this translates into trying to contact a specialist because of the need for information.

Italy was completely unprepared to assist chronic patients with telemedicine, and telemedicine was often implemented only on account of a personal initiative of doctors who were directly contacted by patients (7). In most cases, a simple phone call with a neurologist would reassure patients or help them cope with their situation. We advise services caring for people with chronic diseases such epilepsy to implement some official form of telemedicine. This will reduce the cost of needed follow-up visits and might be suitable for reducing patients' distress during the current new wave of COVID-19. Technology-driven therapies, such as VNS, should facilitate the remote assistance of our patients. Actually, recent developments in VNS technology allow auto-titration to the target intensity and duty cycle (17). Auto-titration changes VNS stimulation parameters at defined intervals; within the therapeutic range, it is usually well-tolerated, and patients feel they have some control since they know on which day the titration will happen. This method makes it possible to reach the target stimulation intensity and duty cycle, avoiding several visits to the outpatient clinic. In the unlikely event that adverse effects (cough, pain, hoarseness) are reported, the patients could temporally stop stimulation using the magnet and consult their neurologist. This feature is very useful, both for clinicians and patients, in periods of reduced disposal of follow-up with a specialist since it spares many ambulatory visits that have the sole purpose of causing a small increase in VNS parameters. We think that a more direct interaction of the patient with VNS devices, such as a patient dedicated app showing the status of stimulation, could help alleviate the preoccupations and anxiety related to the implant. Currently, the only control that a patient has of a VNS device is the possibility to turn on extra-stimulation or temporally stop the device, using a magnetic wristband. Furthermore, remote control for the VNS device (by telephone or internet) is desirable to allow clinicians to intervene in case of urgency (severe adverse effect or catastrophic seizure frequency modification) to modify stimulation parameters.

Present exploratory results, derived from an emergency situation, also offer new ideas to reflect about the protocols of follow-up used for PwE in general. Chronic follow-up of PwE should be mostly guaranteed by a remote service assessing seizure features (frequency, severity, related injuries, post-ictal phenomena), ASM tolerability, quality of life, and psychometric tests and instrumental results (blood, EEG, MRI). As a matter of fact, most PwE with chronic epilepsy do not undergo neurological clinical examination during their controls. Since epilepsy is not usually clinically manifest at the moment of the visit, the neurologist bases his/her decisions mostly on data such as EEG, MRI, and blood tests. All these data can be informatically transferred to the clinician, using coding such as blockchain, which guarantees privacy and traceability of health care data (18), who will reserve the visit only for those cases with critical issues or new problems requiring a physical evaluation. For the rest of PwE, the chronic follow-up might be guaranteed by sporadic physical (yearly) examination.

The present study has some limitations. The small size of the sample reduces its statistical power. Another limitation of our research is that this population, treated with VNS, suffered from a different form of drug-resistant epilepsy, making it hard to reach a homogeneous population, as is done commonly when researchers study a severe form of epilepsy. However, we designed this study to obtain preliminary data for a bigger future multicenter study. Using an online questionnaire did not allow patients with moderate to severe cognitive impairment to answer, and this explains the involvement of caregivers participating in the survey. Anyway, it must be said that the online survey offered the opportunity to reach as many patients as possible during the lockdown phase, allowing the possibility to get in touch with them during isolation. We are aware that the online survey we elaborated on provides low strength of scientific evidence, but it allowed us to understand the needs of patients and caregivers despite the limitations imposed by the lockdown.

## Conclusion

The first wave of the COVID-19 pandemic and the related social restrictions apparently did not impact seizure frequency; however, they caused psychological distress in PwE with implanted VNS. The preliminary findings reported a lack of assistance in patients and showed that many VNS-treated PwE and their caregivers faced problems due to the chronic disease and reported anxiety and depressive symptoms during the pandemic. Disease-related issues were amplified by the lack of telemedicine assistance and the lack of control/information about VNS therapy at the disposal of PwE. These issues should be systematically addressed in order to improve the quality of life of PwE with VNS, especially during periods of lockdown.

## Data Availability Statement

The raw data supporting the conclusions of this article will be made available by the authors, without undue reservation.

## Ethics Statement

Ethical review and approval was not required for the study on human participants in accordance with the local legislation and institutional requirements. The patients/participants provided their written informed consent to participate in this study.

## Author Contributions

GA, TT, and MC designed and conceptualized the study. EG had a major role in the statistical analysis. GA, EG, and JL provided interpretation and wrote the paper. ID, FF, and MS provided database implementation. ID, EG, GB, and MS played a major role in the acquisition of data. MC, GB, GA, TT, and JL revised the manuscript. All authors contributed to the article and approved the submitted version.

## Conflict of Interest

The authors declare that the research was conducted in the absence of any commercial or financial relationships that could be construed as a potential conflict of interest.
