# Supplementary material for: The Impact of COVID-19 Lockdown on People With Epilepsy and Vagal Nerve Stimulation
Source: Front Neurol. 2021 Feb 26;12:640581. doi: 10.3389/fneur.2021.640581 (PMC7952610; doi:10.3389/fneur.2021.640581)
Supplement: Supplementary file 1 [file Data_Sheet_1.docx]

**Supplementary Table 1.** Sex, age, education, marital and working status in the whole sample and in the patients’ sample that underwent to the psychometric questionnaire.

**Supplementary Table 2:** Item in the submitted online survey
